# Comparative study of the chemical composition, antioxidant, and antimicrobial activity of the essential oils extracted from *Lavandula abrialis* and *Lavandula stoechas*: *in vitro* and *in silico* analysis

**DOI:** 10.3389/fchem.2024.1353385

**Published:** 2024-03-25

**Authors:** Mohamed Radi, Zaina Eddardar, Aziz Drioiche, Firdaous Remok, Md. Eram Hosen, Khalid Zibouh, Brahim Ed-Damsyry, Amale Bouatkiout, Sanae Amine, Hanane Touijer, Ahmad Mohammad Salamatullah, Mohammed Bourhia, Samir Ibenmoussa, Touriya Zair

**Affiliations:** ^1^ Laboratory of Innovative Materials and Biotechnology of Natural Resources, Faculty of Sciences, Research Team of Chemistry of Bioactive Molecules and the Environment, Moulay Ismaïl University, Meknes, Morocco; ^2^ Equipe Ecosystèmes et Sciences de l’environnement, Faculté des Sciences Appliquées, Ait Melloul—Université Ibn Zohr, Agadir, Morocco; ^3^ Department of Genetic Engineering and Biotechnology, University of Rajshahi, Rajshahi, Bangladesh; ^4^ Laboratory of Materials, Nanotechnology and Environment, Faculty of Sciences, Mohammed V University in Rabat, Rabat, Morocco; ^5^ Department of Food Science and Nutrition, College of Food and Agricultural Sciences, King Saud University, Riyadh, Saudi Arabia; ^6^ Laboratory of Biotechnology and Natural Resources Valorization, Faculty of Sciences, Ibn Zohr University, Agadir, Morocco; ^7^ Laboratory of Therapeutic and Organic Chemistry, Faculty of Pharmacy, University of Montpellier, Montpellier, France

**Keywords:** lavender, quality control, phytochemicals, antioxidant/antimicrobial activities, oulmès -Morocco, molecular docking

## Abstract

This work aims to add value to the *Lavandula* genus by identifying the chemical composition, antioxidant, and antimicrobial activities of two species lavender from Oulmès in Morocco; *Lavandula abrialis* and *Lavandula stoechas*. The uniqueness lies in the integrated approach that combines *in vitro* and *in silico* analyses to assess the biological properties of the essential oils (EO). The objective of this study is to enhance the significance of the Lavandula genus by analyzing the chemical composition, antioxidant properties, and antimicrobial effects of two lavender species found in Oulmès, Morocco: Lavandula abrialis and Lavandula stoechas. The distinctiveness is in the comprehensive methodology that merges *in vitro* and *in silico* investigations to evaluate the biological characteristics of the essential oils (EO). The extraction of essential oils (EO) by hydrodistillation from the aerial parts of *Lavandula abrialis* gave a high yield of essential oils (2.9%) compared to *Lavandula stoechas* (2.3%). A GC-MS analysis of the chemical composition revealed 56 chemical compounds, with some variation in the predominant components, representing between 99.98% and 100% of the EOs of the studied lavenders. Their antioxidant activity was assessed using the DPPH test. This method revealed that *L. stoechas* EO has a higher percentage of free radical inhibition than *L. abrialis.* The IC_50_ values demonstrate that the antioxidant activity of ascorbic acid is higher (1.62 g/mL) than the EOs of tested plants. Noteworthy, the EO of *L. stoechas* is more potent (12.94 g/mL) than that of *Lavandula tibialis* (34.71 g/mL). Regrading, the antibacterial tests, the EO of *L. abrialis* was particularly active against *Staphylococcus aureus* BLACT, which is inhibited at a concentration of 6.25 g/mL, while *L. stoechas* EO has a strong effect on *Escherichia coli*, with a MIC of 1.56 g/mL. Concerning the antifungal activity of the EOs, yeasts showed sensitivity toward EOs extracted from both *L. tibialis* and *L. stoechas*. Moreover, an *in silico* study was conducted targeting sarA protein of *S. aureus* (PDB ID: 2fnp) and NADPH oxidase from *Lavandula sanfranciscensis* (PDB: 2CDU) and results showed that Ishwarone and Selina-3,7 (11)-diene exhibited highest binding energy with −9.8 and −10.8 kcal/mol respectively. Therefore, these two compounds could be used as an antibacterial and antioxidant agents however more experimental and molecular study should be required.

## 1 Introduction

Aromatic and medicinal plants (MAPs) hold an important place in modern and ancient civilizations (including ancient Egypt and China) and all continents (Fellah et al., 2006). They are used in daily life and consumed for their medicinal and nutritional properties. Increasing demand for MAPs as raw material has been noticed and their use for various purposes in many fields such as pharmacy, agri-food, and phytosanitary sectors (Raal, 2010).

With the increasing resistance of harmful microbes to traditional antibiotics, many scientific studies are now focusing on the use of plant extracts as antibiotic alternatives (Essawi and Srour, 2000; Boutahiri et al., 2022). They are also a rich source of compounds with high antioxidant potential. Indeed, these chemicals protect body cells from free radical damage, as well as maintain food quality and protect it from oxidation (Willcox et al., 2004). In this respect, essential oils have been the focus of various studies to identify new antioxidants of natural origin as an alternative to synthetic antioxidants, which have a certain toxicity (Atmani et al., 2009; Suhaj, 2006), in food preservation, and as a preventative agent for certain human diseases (Saidi et al., 2022; Khettal et al., 2017).

Morocco, due to its geographical area and climatic diversity, is a true phylogenetic reservoir, with approximately 4,200 species, nearly 400 of which belong to 116 families and 726 genera (Fakchich and Elachouri, 2021), allowing it to occupy a privileged position among Mediterranean countries with a long medical tradition and ancestral knowledge and experience on medicinal plants (Scherrer et al., 2005).

The Lamiaceae family, cited by Bachiri et al. (2015), is renowned in the field of herbal medicine for its extensive size, consisting of more than 250 genera and over 7,000 species, as reported by Gourich et al. (2022). Indeed, most of the plants in this family are rich in essential oils and are widely utilized in aromatherapy, perfumery, and the cosmetics industry (Fakchich and Elachouri, 2021; Cox-Georgian et al., 2019; Bekut et al., 2018). Furthermore, it includes the important genus *Lavandula*, which belongs to the subfamily Nepetoideae (Dupont and Guignard, 2012), and contains approximately 39 species, numerous hybrids, and nearly 400 registered cultivars (Upson and Andrews, 2004). This subshrub can grow to be 1 m tall and has blue-purple blooms. White and pink flowers can be found in other varieties (Mohammedi and Fawzia, 2011). In addition to cultivated species, most notably hybrid lavandin, several wild species abound in different regions (Bellakhdar, 1997).

They are aromatic perennials whose medicinal properties are widely used in herbalism and aromatherapy. These therapeutic properties are linked to their primary and secondary metabolites, particularly their essential oils. Indeed, the latter has antispasmodic, antibacterial, antifungal, antioxidant, and acaricidal properties (Mohammedi and Fawzia, 2011; Bachiri et al., 2017). Recent research on lavender essential oils emphasizes that the medicinal properties and fragrance of lavender essential oils are primarily due to their volatile organic compounds. Specifically, monoterpenes and sesquiterpenes are responsible for both the characteristic fragrance of lavender and the therapeutic properties of the essential oils. Moreover, the chemical makeup of the plants’ essential oils is influenced by various factors, including both inherent and external factors such as the genetic makeup, harvesting conditions, drying and preservation methods, and extraction techniques (Bachiri et al., 2016; Bachiri et al., 2017).

The territorial commune of Oulmès, which is part of the mid-Atlas in Morocco, contains several species of the genus *Lavandula*, which has attracted valuation attention due to its large geographic distribution, and represents a very important source of income for the local community, especially farmers and rural women. *Lavandula* has also offered promising prospects in contributing to the sustainable development of this marginal area by organizing producers and collectors into agricultural cooperatives. As well as more than a dozen cooperatives have participated in the improvement and diversification of lavender products, through the design of organic labels and certification of PGI (Protected Geographical Index) and PDO (Protected Designation of Origin) (Provincial Directorate of Agriculture DPA of Khémisset, Monograph of the province of Khémisset, 2022).

In this context, our work is related to the valorization of aromatic and medicinal plants in the plateau of Oulmès through a comparative analysis of the chemical composition, antioxidant activity, and antimicrobial activity of two species of lavender: cultivated lavender (*Lavandula abrialis*) and wild lavender (*Lavandula stoechas*).

## 2 Material and methods

### 2.1 Presentation of the study area

**FIGURE 1:**
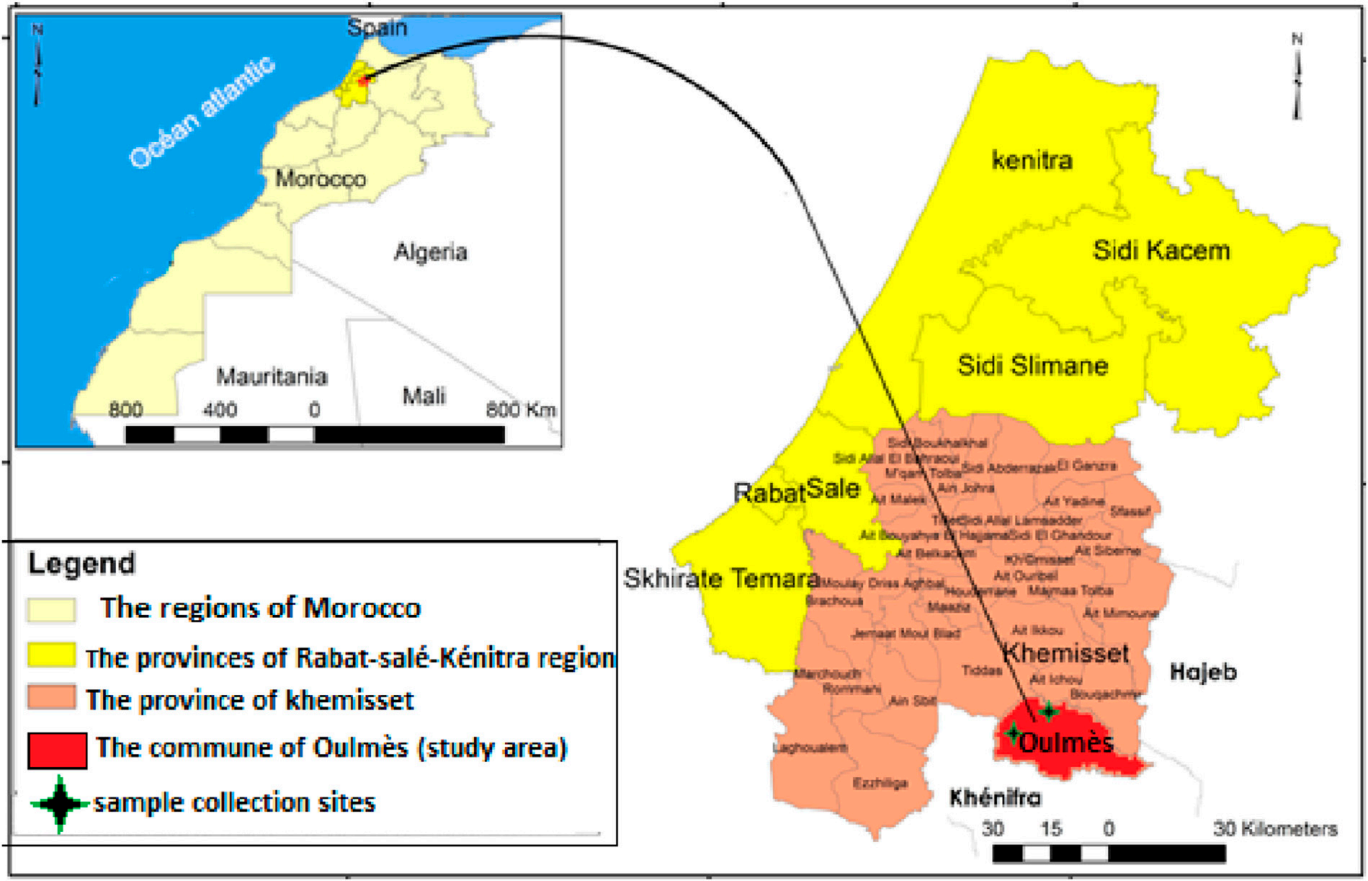
Geographical location of the study area and location of the collected samples.

The commune of Oulmès (Figure 1) is part of the province of Khemisset (Region of Rabat-Sale Kenitra) and it covers an area of 1,001.66 km^2^. This area is dominated by rugged reliefs which represent more than 80% of the total surface area (Provincial Directorate of Agriculture DPA of Khémisset, Monograph of the province of Khémisset, 2022). The climate is subhumid, characterized by wet, cold winters and hot summers. Monthly rains are characterized by a rainfall regime that varies from 1 year to the next, reflecting the irregularity of precipitation with annual averages ranging between 280 and 400. Snowfall can occur from mid-November and the temperature varies between −2 and 40°C (Provincial Directorate of Agriculture DPA of Khémisset, Monograph of the province of Khémisset, 2022). This climate allows the region to be rich in aromatic and medicinal plants and it also contributes to the development of different varieties of lavender.

### 2.2 Plant material

#### 2.2.1 Collection and botanical identification

**FIGURE 2:**
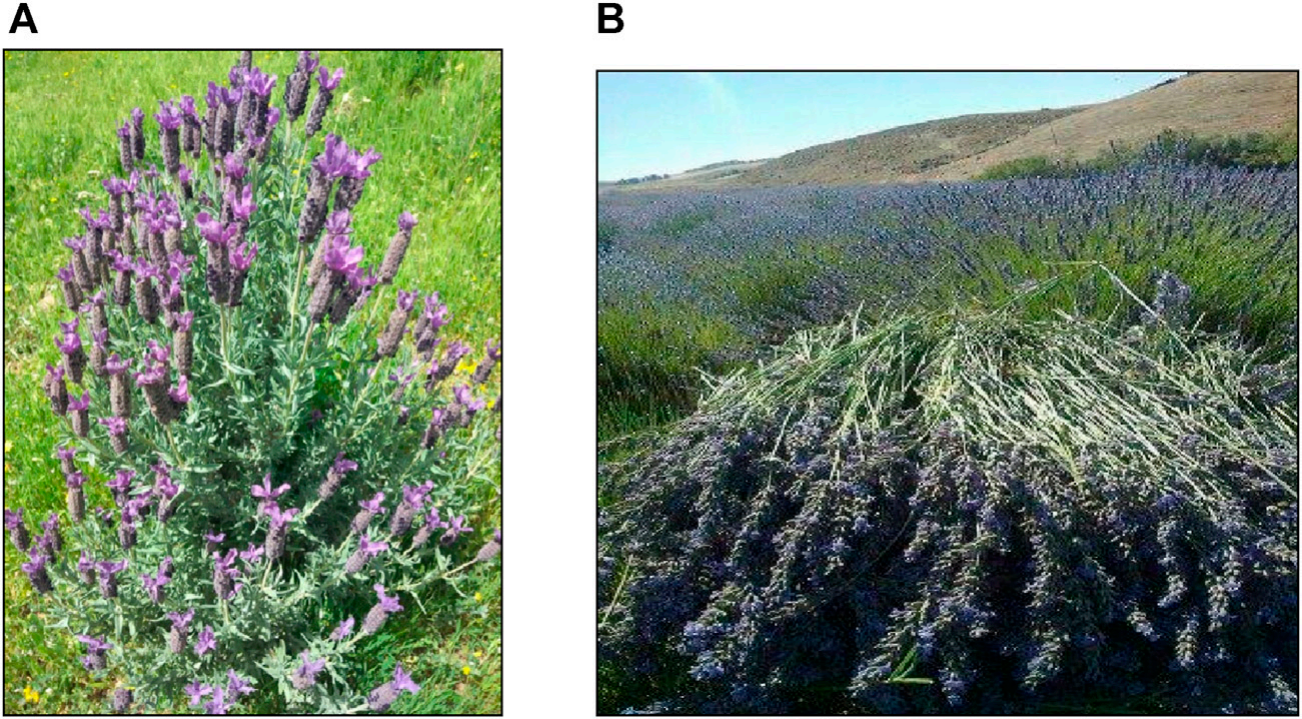
Photos of *L. stoechas* **(A)** *Lavandula abrialis* **(B)** taken by RADI Mohamed and ZAIR Touriya (2022).

*Lavandula abrialis* RAB114629 and *L. stoechas* RAB114630 (Figures 2A, B) were collected in May 2022 in locations listed with their geographical coordinates in Table 1. The botanical identity of these two species was finalized (Table 2) at the Department of Botany, Scientific Institute in Rabat. The samples of leaves and flowers were collected in burlap bags and stored away from light and humidity until needed to preserve the integrity of the molecules as much as possible.

**TABLE 1:** Location of studied species’ collecting sites.

N°	Scientific name	Abbreviation	Collection site	Substrate	Latitude (x)	Longitude (y)	Altitude (m)	Collection year
1	*Lavandula stoechas*	*L. stoechas*	Oulmes	Silice	51°33′8″W	2°58′58″N	750	2022
2	*Lavandula abrialis*	*L. abrialis*	Oulmes	Silice	51°31′30″W	2°52′8″N	810	2022

**TABLE 2:** Botanical classification of two lavenders.

Kingdom	*Plantae*
phylum	*Magnoliophyta*
Class	*Magnoliopsida*
Order	*Lamiales*
Family	Lamiaceae
Subfamily	*Nepetoideae*
Genus	*Lavandula*

### 2.3 Quality control of plant material

#### 2.3.1 Moisture content (MC)

The moisture content of the plant is measured by subjecting 5 g of each plant to a drying process in an oven at a temperature between 100°C and 104°C for a duration of 24 h, following the guidelines outlined in the AFNOR standard (NF - V03-402 1985) (AFNOR NF V03-402, 1985).

The following formula was used to determine the moisture content after each species underwent three repetitions (Equation 1):

$$\text{MC}\%=(\left(m_{i}-m_{f}/m_{i}\right)\times 100$$ (1)

With:

MC: moisture content of the dry plant in %.

m_i_: Initial mass of the plant before drying in (g).

m_f_: Final mass of the plant after drying in (g).

#### 2.3.2 pH determination

The pH of a specific product indicates its level of acidity or alkalinity. A volume of 10 mL of heated distilled water was introduced to 2 g of the experimental specimen. Following agitation, the liquid is subjected to filtration, then cooled, and the pH is determined using a pH meter.

#### 2.3.3 Determination of acidity

Following the protocol, 10 g of the powder from each plant was mixed with 100 mL of distilled water and heated for 15 min while stirring. After the mixture had cooled, it was titrated with a solution of NaOH (N = 0.1) in the presence of phenolphthalein until a persistent pink color developed (AFNOR NF V03-402, 1985).

The acidity index is determined according to the following Equation (2):

$$I_{a}=\left(V\times 56.1\times N\right)/\text{TS}$$ (2)

With:

V: Volume of the KOH solution (0.1 mol/L) in ml.

N: Normality of the KOH solution (0.1 mol/L).

TS: mass of the test sample in g.

56.1: Constant FactorTa.

#### 2.3.4 Ash content

According to AFNOR NF V05-113 standard, 1972 (AFNOR NF V05-113,1972), the determination of the ash content is based on the destruction of any carbon particle of a 2 g sample of each plant, under a temperature of 550°C. The operation will only be completed when the color of the residue becomes grayish-white, which will turn into a white color after cooling.

Organic matter is calculated according to the following Equation (3):

$$\text{OM}\%=\left(\left(W_{1}-W_{2}\right)/\text{TS}\right)\times 100$$ (3)

With:

OM%: Organic matter.

W_1_: Weight of the capsule and the sample before calcination.

W_2_: Weight of the capsule and the sample after calcination.

TS: test sample.

The ash content (or mineral matter content: MM) is determined as follows: MM = 100-OM%

#### 2.3.5 Determination of heavy metals by induced plasma coupled atomic emission spectrometry (ICP-AES)

To determine the content of seven heavy metals (As, Cd, Cr, Fe, Pb, Sb, and Ti) in the plant matter of studied plants, the standardized mineralization protocol (NF EN, 1999-1-1/A1, 1999) was used.

This process consists of mixing the crushed plant material (0.1 g) with 3 mL of aqua regia prepared from 1 mL of nitric acid HNO_3_ (99%) and 2 mL of hydrochloric acid HCl (37%), The mixture refluxes at 200°C for 2 hours. Once liquid is chilled and separated from solid particles, it is collected and filtered through a 0.45 µm pore size filter. The mixture is diluted with distilled water to 15 mL. The concentrations of heavy metals are determined by the inductively coupled plasma atomic emission spectrophotometer ICP-AES (Ultima 2 Jobin Yvon) at the TSUSR laboratory (Technical Support Unit for Scientific Research) at CNRST in Rabat (Skujins, 1998).

### 2.4 Yields, characterization, and quality control of EOs

#### 2.4.1 Extraction and determination of EO yields

The essential oils of the two lavenders were obtained using hydrodistillation using a Clevenger-type device (Clevenger, 1928). A mass of 100 g of plant material, of each dried and mixed lavender, is submerged in water (1 L) in a 2 L flask. This mixture was boiled for 3 h at a temperature of 90°C. Each plant material underwent three cycles. The oil was dehydrated using anhydrous sodium sulfate (Na2SO4) and then kept in an airtight brown glass container at a temperature of 4°C until it was ready to be used.

The EO Yield was calculated according to the following formula (Equation 4):

$$\text{Yield }\left(\%\right)=\left(V\left(\text{EO}\right)\right)/M_{0}\times 100$$ (4)

With:

V (EO): Volume of EO recovered (g).

M_0_: Mass of plant material (100 g).

#### 2.4.2 Analysis and identification of the chemical composition of EOs

The analysis of the lavender EO samples was carried out by a Thermo Electron type gas chromatograph (Trace GC Ultra) coupled to a Thermo Electron Trace MS system mass spectrometer (Thermo Electron: Trace GC Ultra; Polaris Q MS). Fragmentation is carried out by electronic impact with an intensity of 70 eV. The chromatograph is equipped with a DB-5 column (5% phenyl-methyl-siloxane) (30 m × 0.25 mm x 0.25 μm film thickness), and a flame ionization detector (FID) powered by a mixture of H_2_/Air gas. The column temperature is programmed at a rate of 4°C/min from 50°C to 200°C for 5 min. The device has a split–splitless PVT (Programmed Vaporization Temperature) injector. Split injection is employed, with a leakage ratio of 1/70 and a flow rate of 1 mL/min for the vector gas nitrogen.

The identification of the constituents of essential oils was made based on the determination and comparison of the Kovats indices (KI) of the compounds with those of the standard products known and described in the databases of Kovats (1965) and Adams (2007), By conducting a comparison of the peak retention periods with the known legitimate standards present in the authors’ laboratory, as well as comparing the stated KI and MS data with the mass spectral database standards of WILEY and NIST 14, along with published literature.

### 2.5 Quality control of EO

#### 2.5.1 Density

The density of an essential oil is determined by using a pycnometer at a temperature of 20°C. The term refers to the ratio between the density of the oil and the density of pure water at the same temperature. The determination was made based on the following formula (Equation 5).

$$d_{20}=\left(m_{2}-m_{0}\right)/\left(m_{1}-m_{0}\;\right)$$ (5)

With

m_0_: Mass in grams of the empty pycnometer.

m_1_: Mass in grams of the pycnometer filled with water.

m_2_: Mass in grams of the pycnometer filled with oil.

#### 2.5.2 Acid value

The acid value is the number of mg of KOH necessary for the neutralization of the free acids contained in 1 g of EO. The free acids are neutralized with a titrated ethanolic solution of potassium hydroxide (KOH). In our case, we used a 0.1 N KOH solution. Phenolphthalein is used as a color indicator.

The acidity result is expressed in % oleic acid by the following formula (Equation 6):

$$\text{Acidity }\%=\left(V^{*}N^{*}M_{A}\right)/\left(10^{*}\text{TS}\right)\text{ and therefore},\text{acid value}=\left(N^{*}\left(V-V_{0}\right)\;*M_{\text{KOH}}\right)/\text{TS}$$ (6)

With:

V: Buret drop volume (in ml).

N: Normality of the KOH solution.

TS: Test sample in g.

M_A_: Molecular weight of oleic acid = 282 g/mol.

#### 2.5.3 Iodine value

The Iodine value (IV) is a crucial analytical feature of oil that serves as an indication of its unsaturation. It is calculated according to the ISO 3961 method, by determining the number of grams of iodine attached to the double bonds present in 100 g of fat.

A 0.1 g quantity of the oil is added to a 100 mL Erlenmeyer flask along with 4 mL of chloroform and 5 mL of iodine monochloride (Wijs reagent). The solution is agitated gently and then stored in a dark location for 1 h at a temperature of 20°C. Subsequently, 4 mL of a potassium iodide solution with a concentration of 10% and 30 mL of distilled water are introduced.

The resulting mixture is titrated with sodium thiosulfate (0.1 N) using a few drops of starch solution as a colored indicator. The titration is continued until the blue color disappears. A blank test without oil is conducted at the same time and under the same circumstances.

The results are expressed as follows (Equation 7):

$$\text{IV}=\left[\text{gde }I_{2}/100\text{ gd}’\text{oil}\right]=\left(126.9*N*\left(V_{0}-V_{1}\right)\right)/\text{TS}$$ (7)

With:

IV: Iodine value.

V_0_: Volume of sodium thiosulfate used for the blank titration.

V_1_: Volume of the sodium thiosulfate solution.

N: Normality of the thiosulfate solution.

TS: Test sample.

#### 2.5.4 Peroxide value

Peroxide value (PV) is the most widely used chemical technique for evaluating the oxidative degradation of unsaturated fatty acids in essential oils.

According to regulation CEE/2568/9, the prescribed amounts for the combination are as follows: 0.5 g of oil in a container with a volume of 100 mL, 4 mL of dichloromethane, 6 mL of acetic acid, and 0.1 mL of an aqueous solution containing saturated potassium iodide (15 g of potassium iodide diluted in 10 mL of distilled water). Afterward, the bottle is tightly sealed and forcefully shaken for a period of 1 min. Afterwards, the balloon is left undisturbed in darkness for 5 min, and then 10 cc of distilled water is added.

The liberated iodine is quantitatively determined by aggressively swirling it with a 0.1 N thiosulfate solution in the presence of starch as a chromogenic indicator. A control experiment, devoid of any oil, is conducted simultaneously and under identical circumstances.

The peroxide index (PI) is expressed in milliequivalents of oxygen per kg of oil (Equation 8).

$$\text{PV }\left[M_{\text{eq}}\;O_{2}/\text{Kg}\right]=\left(V^{*}1000^{*}N\right)\;/\text{ST}$$ (8)

With:

V: Volume poured with sodium thiosulfate (in ml).

TS: Oil sample to be analyzed in g.

N: Normality of sodium thiosulfate solution.

### 2.6 Antioxidant activity of essential oils by DPPH radical test

This test aims to evaluate the anti-radical effect of EOs from the plants studied using a stable free radical which is 2,2-diphenyl-1-picrylhydrazyl (DPPH) (Wong et al., 2006).

The DPPH solution is prepared by solubilizing 2.4 mg of DPPH• in 100 mL of pure methanol. The test is carried out by mixing 100 µL of each EO with 3.9 mL of DPPH• solution. The standard used is ascorbic acid at different concentrations. At the same time, a negative control (blank) was carried out with pure methanol alone. All tests are repeated three times. The samples are then left in the dark for 30 min, and the discoloration compared to the negative control containing only the DPPH• solution is measured at 517 nm.

The results were expressed as percentage reduction or inhibition of DPPH•(AA%) using the following formula (Equation 9):

$$\text{AA}\%=\left(A_{\text{control}}-A_{\text{sample}}\right)/A_{\text{control}}\times 100$$ (9)

With:

AA%: Percentage of antioxidant activity.

A _control_: Absorbance of the solution containing only the solution of the DPPH• radical.

A _sample_: Absorbance of the solution of the samples to be tested in the presence of DPPH•.

The study of the variation in absorbance as a function of the concentration of ascorbic acid made it possible to determine the inhibitory concentrations of EOs (IC_50_), the values of which were determined graphically. It should be noted that since there is no absolute measure of the antioxidant capacity of a compound, results are often compared to a reference antioxidant, such as ascorbic acid.

### 2.7 Antimicrobial activity

#### 2.7.1 Biological material

The assessment of the antibacterial efficacy of the essential oils (EOs) derived from two types of lavender was conducted on a total of 10 microorganisms (Table 3). All strains (bacterial and fungal) were isolated from the hospital environment: Mohamed V Provincial Hospital-Meknes. These specific microbes are pathogenic, renowned for their robust antibiotic resistance, as well as their ability to invade and produce toxins in humans. These strains were taken from a 20% glycerol stock at −80°C, rejuvenated on Mueller Hinton and Sabouraud broths, and subcultured before use (Ailli et al., 2023).

**TABLE 3:** List of bacterial strains tested with their references.

Strains			References	Strains			References
Bacteria	Gram-positive Cocci	*Staphylococcus epidermidis*	5,994	Fungal	Yeasts	*Candida albicans*	Ca
		*Staphylococcus aureus* BLACT	4IH2510			*Candida dubliniensis*	Cd
	Gram-negative Bacilli	*Escherichia coli sauvage*	3DT1938			*Candida parapsilosis*	Cpa
		*Enterobacter cloacae*	02EV317			*Candida tropicalis*	Ct
		*Klebsiella pneumoniae*	3DT1823		Molds	*Aspergillus niger*	AspN

#### 2.7.2 Determination of the minimum inhibitory concentration, the minimum bactericidal concentration, and the minimum fungicidal concentration

The minimum inhibitory concentration (MIC) is defined as the lowest concentration of essential oil capable of inhibiting the growth of a microorganism (Kuete,2 004). The determination of the MIC was carried out using the microdilution method (Balouiri et al., 2016; Ailli et al., 2023). The MIC value of each extract was determined using a sterile 96-well microtiter plate. A microtiter plate was prepared by transferring 100 µL of Mueller-Hinton broth for bacteria and Sabouraud for fungi to all wells. From a stock solution of the essential oil prepared in 10% DMSO, a dilution series was carried out to obtain concentrations of 5 to 0.93 10^−2^ mg/mL of each EO. And then, 10 μL of inoculum 10^6^ CFU/mL (bacteria) or 10^4^ CFU/mL (fungi) was added to all wells. The microplates were incubated for 24–48 h at 37°C. After incubation, 10 µL of resazurin (5 mg/mL) is added to each well as an indicator of microbial growth. After a second incubation for 2 h at 37°C, microbial growth was revealed by the change in color from purple to pink. The MIC value is determined as the lowest concentration that prevents a color change of resazurin. To determine the minimum bactericidal concentration (MBC)/minimum fungicidal concentration (MFC), 10 µL was taken from each well without visible growth and inoculated in Mueller Hinton (MH) agar for bacteria or in Sabouraud for fungi. The boxes are incubated for 24 h at 37°C. The MBC and MFC were defined as the lowest concentrations of the analyzed samples that produced a 99.99% reduction in UFC/mL compared to the control (Bachiri et al., 2016). The MBC/MIC or MFC/MIC ratio is calculated to evaluate the antimicrobial potency. If this ratio is less than 4, the effect of the EO is bactericidal/fungicidal, if the ratio is greater than 4, the sample has a bacteriostatic/fungistatic effect.

### 2.8 In silico studies

#### 2.8.1 Ligand preparation

The GC-MS analysis detected total 56 phytochemicals from *L. stoechas* and *L. abrialis* were used as a ligand for docking analysis. For virtual screening, all ligands are downloaded from PubChem (https://pubchem.ncbi.nlm.nih.gov/) in SDF format.

#### 2.8.2 Protein preparation

For *in silico* antibacterial and antioxidant analysis, the x-ray crystal structure of sarA protein of *Staphylococcus aureus* (PDB ID: 2fnp) and NADPH oxidase from *Lavandula sanfranciscensis* (PDB: 2CDU) were retrieved from the protein data bank (https://www.rcsb.org/). The Discovery Studio software was employed to clean the structure of the selected proteins obtained from the Protein Data Bank. Heteroatoms were eliminated during this process. Subsequently, utilizing the GROMOS96 43b1 force field and the SwissPDB Viewer software, the energy of the refined protein structures was subjected to minimization and optimization procedures.

#### 2.8.3 Molecular docking study

The molecular docking analysis involving the interaction of phytochemicals derived from *L. stoechas* and *L. abrialis* with target proteins was performed using the PyRx software through the autodock wizard. The protein structure underwent conversion into a macromolecule, while the ligands were transformed into the PDBQT format. For the docked complexes, the center and grid box size were as on their binding pocket. The final docking calculations were executed using PyRx, and the selection of top molecules was based on lower binding energy. Subsequently, Discovery Studio software was employed to investigate the binding interactions and poses of the docked complexes.

### 2.9 Statistical analyzes

Results were presented using the mean ± standard mean error. Statistical significance was set at p<0.05 . To highlight the relationships and correlations of antioxidant and antimicrobial activities with the majority of compounds detected in the essential oils of the two species studied, a heat map was developed using R Software (version 4.1.3).

## 3 Results and discussion

### 3.1 Quality control of lavenders plant material

The collected samples underwent quality control by assessing several distinguishing factors, including moisture content (MC), pH, acidity, ash, and heavy metal content. The results are presented in Tables 4 and 5.

**TABLE 4:** Quality control of plant matter: moisture content (MC), pH, acidity, organic material (OM), and mineral matter (MM).

Species	MC (%)	pH	Acidity (%)	OM (%)	MM (%)
*L. stoechas*	26.26 ± 0.11	5.94 ± 0.01	0.14 ± 0.02	91.83	8.17
*L. abrialis*	20.10 ± 0.26	4.96 ± 0.02	0.11 ± 0.04	91.04	8.96

**TABLE 5:** Concentration of heavy metals (mg/L) (ICP) and FAO/WHO limit value (2009).

Espèce	Chrome (Cr)	Antimony (Sb)	Lead (Pb)	Cadmium (Cd)	Iron (Fe)	Titanium (Ti)
*L. stoechas*	0.0074	0.0154	0.0067	Undetectable	4.525	0.025
*L. abrialis*	0.0217	0.302	Undetectable	Undetectable	2.945	0.0406
limit value	2	1	3	0,3	20	–

#### 3.1.1 Determination of moisture content of lavender plant material

The water content of *L. stoechas* and *L. abrialis* varied between 26.26% and 20.10% respectively. Several factors could influence the moisture content of plants such as the nature of the fibers, the age of the plants, the condition of the soil, and the shelf life of the plant after harvest (Bachiri et al., 2016).

#### 3.1.2 Determination of pH and acidity of lavenders plant material

The hydrogen potential provides information regarding the assimilation of nutrients by plants (Saidi et al., 2023). In the present work, the pH of L. *abrialis* and *L. stoechas* ranged from 4.96 to 5.94, respectively. These values allowed these two lavenders to absorb nutrients such as iron, which was confirmed by the results of the ICP analysis.

#### 3.1.3 Ash content of lavenders plant material

The analyses performed on studied samples showed that the amounts of organic matter in *L. stoechas* and *L. abrialis* were approximately 91.83% and 91.04%, respectively. However, the amounts of mineral matter (or ash) were low, coming in at only 8.17% and 8.96%, respectively (Table 4). These findings showed that organic matter represented most of the weight of these plants.

#### 3.1.4 Determination of heavy metals in lavenders plant material by induced plasma coupled atomic emission spectrometry (ICP-AES)

Atomic absorption spectrophotometry is used to determine the concentration of heavy metals (Cr, Sb, Pb, Cd, Fe, and Ti) in two studied lavender plants. According to the results shown in Table 5, several metals were undetectable and had very low and fluctuating quantities. Additionally, the analyzed samples had values that were below the FAO/WHO (2009) limit threshold.

### 3.2 Yield, characterization, and quality control of lavender EOs

#### 3.2.1 Yield of essential oils from lavenders

Hydrodistillation was used to extract the essential oils from the aerial parts of *L. stoechas* and *L. abrialis*. The essential oils produced ranged in color from light yellow to clear yellow but were always extremely strongly scented.

The yield of essential oils varies greatly from one species to another, according to the results obtained in Table 6, we noted that *Lavandula abrialisa* gave a good yield of essential oil (2.9% ± 0.13%) and *L. stoechas* revealed almost 2.3% ± 0.11%.

**TABLE 6:** Yield of essential oils (EO).

Species	EO yield (%)	Color	Smell
*L. stoechas*	2.3% ± 0.11%	Light yellow	Strong
*L. abrialis*	2.9% ± 0.13%	Clear yellow	Strong

EO, essential oils.

These findings corroborate those of Bachiri et al. (2016) and N'Dédianhoua et al. (2014), who also used hydrodistillation to recover the aerial parts of lavender plants from Morocco’s Mid-Atlas region. However, research on *L. stoechas* from Turkey (Ahmet et al., 2002) and Algeria (Mohammedi and Fawzia, 2011). found that their respective EO yields were low at 1.33% and 1.2%. The essential oil content of *L. abrialis* was slightly higher than the findings of Zrira (2007). Various factors, such as the environment, genotype, geographical origin of the plant, cultural practices, and extraction method, may contribute to differences in essential oil production between species (Aberchane et al., 2001; Bourkhis et al., 2009).

#### 3.2.2 Analysis and identification of the chemical composition of lavender EOs

The GC/MS analysis of the essential oil of *L. abrialis* revealed 56 chemical compounds, totaling 99.98% (Table 7), including 72.13% oxygenated monoterpenes, 18.59% oxygenated sesquiterpenes, 7.7% hydrocarbon monoterpenes, and 1.56% sesquiterpenes (Figure 5A).

**TABLE 7:** Chemical composition of *L.stoechas* and *Lavandula abrialis* EOs.

N°	IK	Compounds	*L. stoechas*	*L.abrialis*
1	926	Tricyclene	0.45	-
2	939	α-Pinene	7.19	0.76
3	954	Camphene	6.07	0.63
4	975	Sabinene	-	0.28
5	979	β-Pinene	-	0.54
6	990	Myrcene	-	1.09
7	1,009	Hexyl acetate	-	0.33
8	1,026	ο-Cymene	-	0.97
9	1,029	Limonene	2.56	1.51
10	1,031	1,8-Cineole	1.53	9.28
11	1,037	(Z)-β-Ocimene		0.93
12	1,059	γ-Terpinene	0.44	0.52
13	1,072	cis-Linalool oxide	0.64	1.06
14	1,086	Fenchone	9.58	-
15	1,088	Terpinolene	-	0.47
16	1,086	trans-Linalool oxide	-	1.36
17	1,096	Linalool	3.27	19.16
18	1,116	endo-Fenchol	1.26	-
19	1,126	α-Campholenal	0.77	-
20	1,146	Camphor	24.35	11.24
21	1,169	Borneol	4.45	9.88
22	1,177	Terpinen-4-ol	0.89	2.63
23	1,182	ρ-Cymen-8-ol	0.48	0.43
24	1,188	α-Terpineol	-	5.99
25	1,192	Hexyl butanoate	-	1.04
26	1,193	Dihydro carveol	0.78	-
27	1,205	Verbenone	1.44	-
28	1,216	trans-Carveol	0.49	-
29	1,229	Nerol	-	0.84
30	1,257	Linalool acetate	-	13.41
31	1,252	Geraniol	-	2.12
32	1,285	Bornyl acetate	1.33	-
33	1,290	Lavandulyl acetate	-	2.88
34	1,326	Myrtenyl acetate	0.48	-
35	1,332	Hexyl tiglate	-	0.47
36	1,351	α-Cubebene	0.51	-
37	1,361	Neryl acetate	-	1.44
38	1,381	Geranyl acetate	-	2.45
39	1,419	(E)-Caryophyllene	-	1.06
40	1,456	(E)-β-Farnesene	-	0.50
41	1,481	Germacrene D	0.78	
42	1,490	β-Selinene	1.24	
43	1,509	Lavandulyl isovalerate	-	0.34
44	1,523	δ-Cadinene	0.57	-
45	1,522	trans-Calamenene	1.24	-
46	1,496	Valencene	0.49	-
47	1,546	Selina-3,7 (11)-diene	2.11	-
48	1,583	Caryophyllene oxide	-	0.38
49	1,587	Gleenol	0.94	-
50	1,640	epi-α-Cadinol	2.18	1.66
51	1,646	Cubenol	15.54	-
52	1,681	Ishwarone	0.99	-
53	1,654	α-Cadinol	1.08	-
54	1,599	Widdrol	0.63	-
55	1,685	α-Bisabolol	1.42	1.97
56	1,689	cis-14-nor-Muurol-5-en-4-one	1.83	-

IK, index kovats.

**FIGURE 3:**
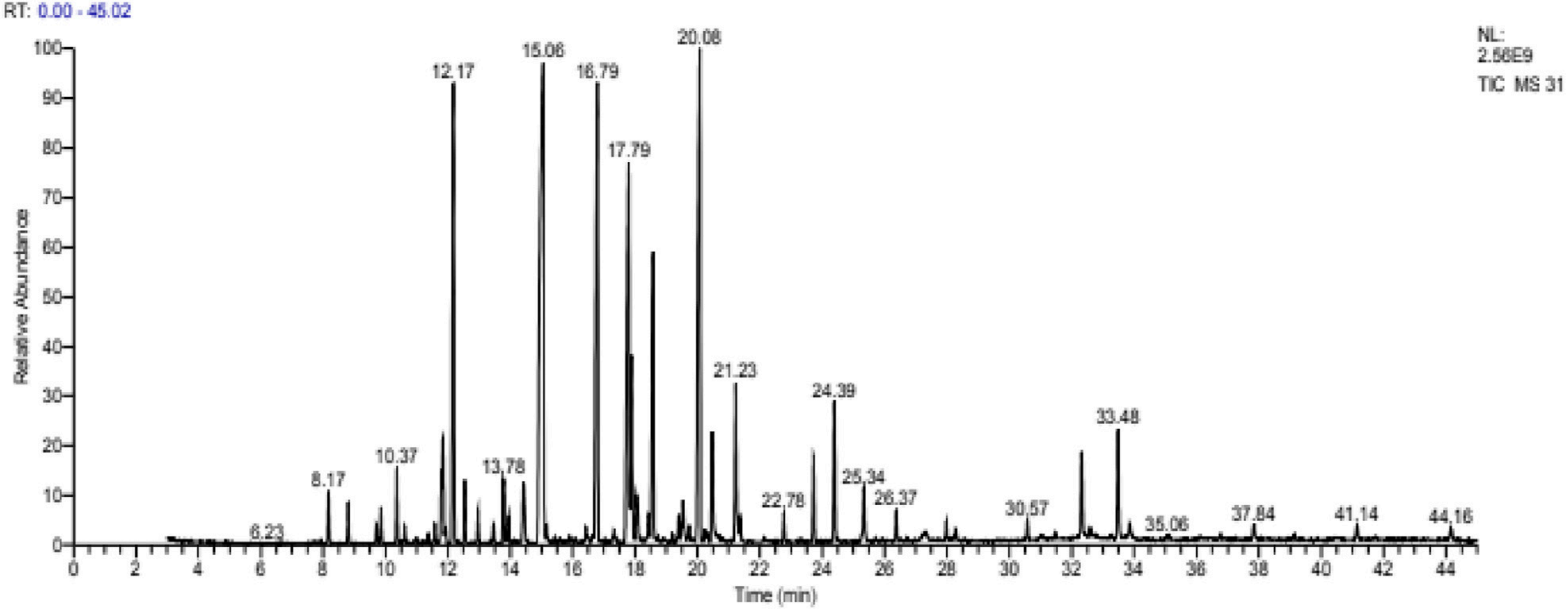
Chromatogram of *Lavandula abrialis*.

The main volatile compounds of the essential oil extracted from the flowers and leaves of *L. abrialis* (Figure 3) were linalool (19.16%), linalool acetate (13.41%), camphor (11.24%), borneol (9.88%), and cineole <1.8->(9.28%). This EO also contained other compounds such as terpineol<α-> (5.99%), lavandulylacetate (2.88%), terpinen-4-ol (2.63%), geranylacetate (2.45%) and geraniol (2.12%).

Our results corroborate with studies carried out in Mediterranean areas with small differences in the percentages. N’Dédianhoua et al. (2014) showed that linalool (25.86%), linalool acetate (13.66%), camphor (16.06%), borneol (11.94%), and Cineole<1.8-> (16.04%) were the main components of *L. abrialis* EO collected in the Mid-Atlas of Morocco. Zrira (2007) mentioned that the EO of this species is dominated by linalool (33.7%), camphor (17.6%), cineole <1.8> (14.5%) and linalylacetate (13.5%).

**FIGURE 4:**
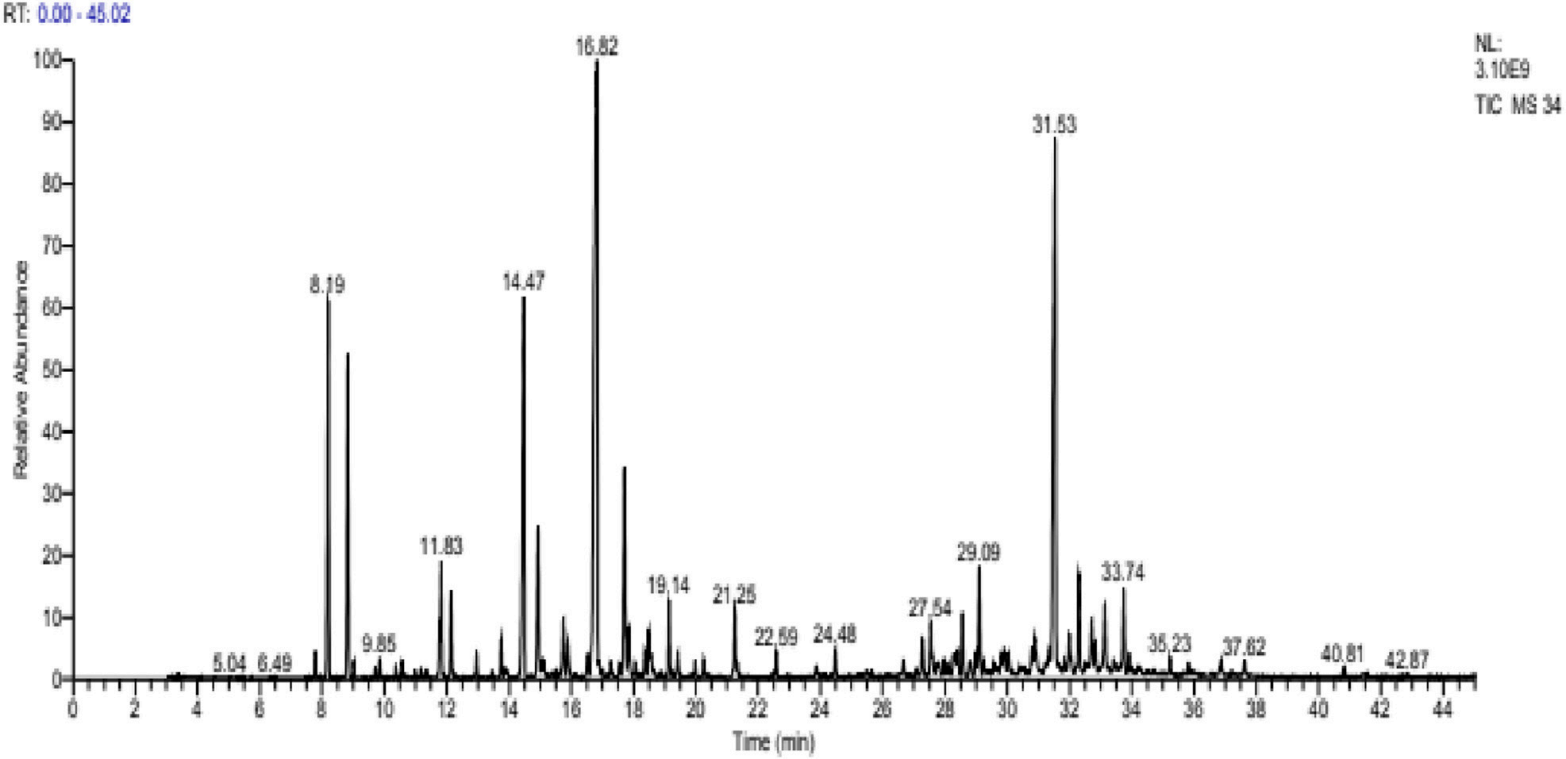
Chromatogram of *Lavandula stoechas*.

**FIGURE 5:**
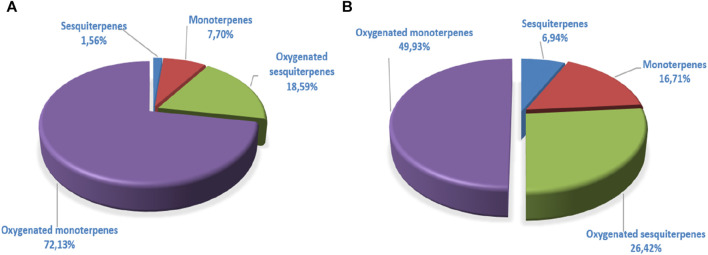
Distribution of terpene families of *Lavandula abrialis* **(A)** and *Lavandula stoechas* **(B)** Eos.

The analysis of *L. stoechas* (Figure 4) EO also showed 56 compounds. The latter represents 100% of its total composition (Table 7). The main components of this oil were camphor (24.35%), cubenol (15.54%), fenchone (9.58%), Pinene<α-> (7.19%), camphene (6.07%) and borneol (4.45%). The compounds identified were classified into four chemical families, namely, oxygenated monoterpenes (49.93%), oxygenated sesquiterpenes (26.42%), monoterpenes (16.71%) and sesquiterpenes (6.94%) (Figure 5B).

Previous studies have noted a distinct chemical composition. According to Mohammedi and Fawzia (2011), the EO of *L. stoechas* collected in Telmsen, Algeria, is distinguished by the presence of 21 compounds, including camphor (18.1%), cineole (18.9%), and fenchone (27.6%). Our sample had different contents, according to the chemical analysis than those examined by Ahmet et al. (2002) for Turkish *L. stoechas*, where they discovered high concentrations of pulegone (40.37%), hexahydrothymol (menthol), and menthone (12.57%).

**FIGURE 6:**
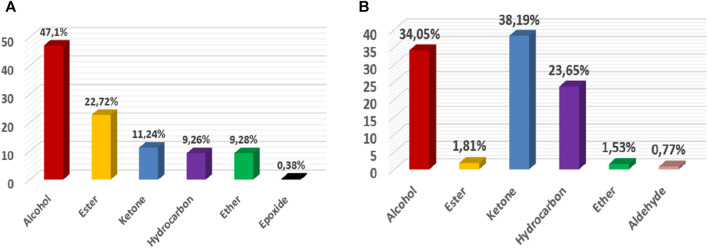
Distribution of families of chemical compounds of EOs of *Lavandula abrialis* **(A)** and *Lavandula stoechas* **(B)**.

A comparison of the chemical composition of the EOs demonstrated that these two lavenders contained variable proportions of oxygenated monoterpenes and oxygenated sesquiterpenes. The EO of *L. abrialis* was rich in alcohol (47.1%) followed by esters (22.72%) and ketones (11.24%) (Figure 6A), while the EO of *L. stoechas* was dominated mainly by ketones (38.19%), alcohols (34.05%), and hydrocarbons (23.65%) (Figure 6B). In addition, other chemical families were found in the EOs of these plants but with low percentages, specifically ethers, aldehydes, and epoxides.

### 3.3 Quality control of lavenders EOs

#### 3.3.1 Density of EOs

*L.stoechas* and *L. abrialis* both produced EOs with nearly identical densities. It fluctuated between 0.925 ± 0.001 and 0.929 ± 0.002 g/mL, respectively. These two EOs can be characterized as light oils because of their lower density (0.9982 g/mL) than water. The density of these oils complied with the AFNOR standard 2005 which sets a density of 0.906–0.990 as quality references (AFNOR, 2005; De Cliff and Harerimana, 2013).

#### 3.3.2 Acid value of EOs

The acid value indicates the content of free fatty acids in studied EOs. A low acidity value characterizes the purity and stability of an oil at room temperature [14]. The EOs of *L. stoechas* and *L. abrialis* had acid values between 10.09 ± 0.01 and 4.49 ± 0.003 respectively. These values indicate that *L. stoechas* oils were more susceptible to undergo alterations than *L. abrialis* EO.

#### 3.3.3 The iodine value of EOs

The iodine value indicates the overall degree of unsaturation of the oils. The more unsaturated an oil is, the higher its iodine value (Wolff, 1968). The values of the iodine values of the EOs of the studied species (0.647 ± 0.01 for *L. abrialis* and 0.546 ± 0.01 for *L. stoechas*) were lower than those provided by the Codex Alimentarius standard (87–111 g of iodine/100 g) (Codex Alimentarius, 2017), and thus the conservation of these EOs could be done without running a significant risk of auto-oxidation.

#### 3.3.4 Peroxide value of EOs

The peroxide value is studied to assess the oxidation state of essential oils. The peroxide value of *L. stoechas* EO (18 ± 0.2) was higher than that of *L. abrialis* essential oil (14 ± 0.1). Comparing these values with those of the commercial standards CODEX STAN 210-1999, the two oils were categorized as good since they match the standards.

### 3.4 Antioxidant activity of essential oils by the DPPH anti-radical method

**FIGURE 7:**
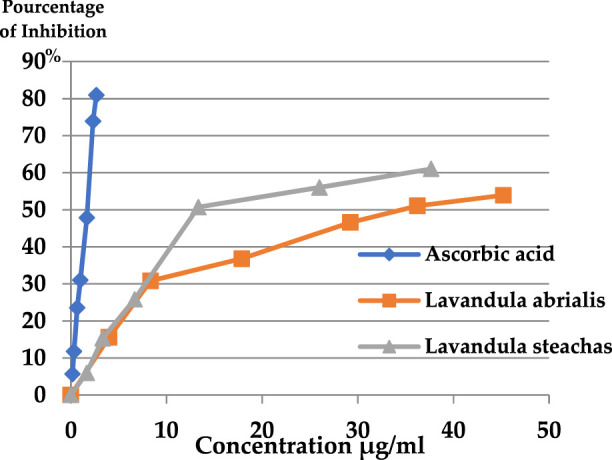
Percentage of DPPH inhibition of the EOs studied.

**FIGURE 8:**
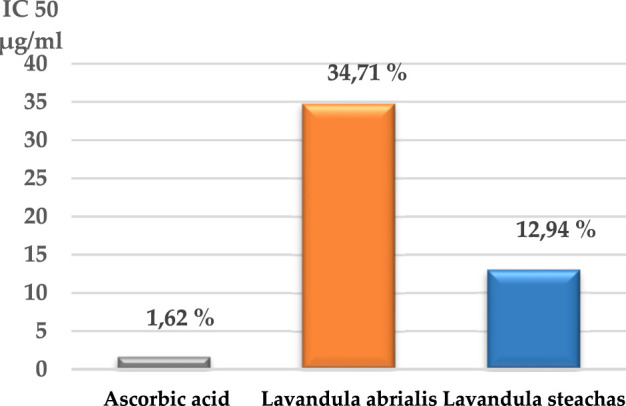
IC_50_ of the EOs of studied lavender.

The antioxidant activity of the essential oils of the two lavenders was evaluated using the DPPH (2,2-diphenyl-1-picrylhydrazyl) test. The obtained results are displayed in Figure 7. The capacity of DPPH radicals to be scavenged was measured in terms of the percentage of DPPH free radicals that were inhibited by the antioxidants found in the various essential oils under study. The best antioxidant activity was indicated by the highest percentage of inhibition. Concentrations that provide 50% inhibition (IC_50_) were calculated from the curve in Figure 7 and are presented in Figure 8.

According to the present findings, the percentage of inhibition of free radicals increases with the increase in the concentration of ascorbic acid or EOs of the two lavenders. Furthermore, *L. stoechas* essential oil exhibited greater antioxidant activity than *L. abrialis* (61.03% and 53.93%, respectively) and lower than ascorbic acid (80.95%). The IC_50_ values showed that ascorbic acid has a higher antioxidant power (1.62 μg/mL) than EOs of the two studied lavenders and that *L. stoechas* EO was more effective (IC_50_ = 12.94 μg/mL) than *L. abrialis* EO (IC_50_ = 34.71 μg/mL). This antioxidant activity of extracted oils may have a connection with the chemical constituents that compose them. Indeed, camphor which is a major compound of the two studied EOs (24.35% for *L. stoechas* and 11.24% for *L. abrialis*)*,* presented a strong correlation with the antioxidant activity, which was confirmed by the results of the heat map. Previous studies have confirmed that this compound also has strong antioxidant activity (Radi et al., 2022; Laib, 2012). Additionally, to camphor’s particular antioxidant properties, the synergistic interactions that can be established between the different constituents of essential oil were also behind its considerable antioxidant power (Laib and Barkat, 2011; Radi et al., 2022; Laib, 2012; Fatiha et al., 2011).

### 3.5 Antimicrobial activity of lavender EOs

The assessment of the antibacterial activity of the studied lavender EOs was carried out at different concentrations on five bacterial strains (Table 8) (three Gram-negative bacteria and two Gram-positive bacteria). Table 9 shows that the EOs extracted from studied plants exhibit significant bactericidal activities against all bacteria tested. Indeed, these EOs inhibited the growth of the strains; Wild *Escherichia coli*, *S. aureus* BLACT, and *Enterobacter cloacae* at an amount of 1.56–12.5 μg/mL. For the two other strains (*Klebsiella pneumoniae* and *Staphylococcus epidermidis*), growth was inhibited at a concentration of 25 μg/mL.

**TABLE 8:** MIC and MBC values (µg/mL) of the studied essential oils (µg/mL).

	*L. abrialis*		*L. stoechas*	
	MIC	MBC	MIC	MBC
*Enterobacter cloacae*	12.5	12.5	12.5	12.5
*Klebsiella pneumoniae*	25	25	25	25
*Escherichia coli sauvage*	12.5	25	1.56	3.13
*Staphylococcus aureus* BLACT	6.25	6.25	12.5	25
*Staphylococcus epidermidis*	25	25	25	25

MIC, minimum inhibitory concentration; MBC, minimum bactericidal concentration.

**TABLE 9:** MIC (µg/mL) of antibiotics evaluated by BD Phoenix for selected species.

Microorganism	References	MIC(µg/mL) identification instrument and antibiogram BD Phoenix™			
		Gentamicin	Amoxicillin-clavulanate	Vancomycin	Trimethoprim-sulfamethoxazole
*Staphylococcus epidermidis*	5,994	2		>8	>4/76
*Staphylococcus aureus BLACT*	4IH2510	<0,5		2	<10
*Escherichia coli sauvage*	3DT1938	2	8/2		≤1/19
*Escherichia coli BLSE*	2DT2057	2	>8/2		>4/76
*Enterobactercloacae*	02EV317	>4	>8/2		>4/76
*Klebsiellapneumoniae*	3DT1823	≤1	≤2/2		≤1/19
*Proteus mirabilis*	2DS5461	2	≤2/2		>1/19
*Pseudomonas aeruginosa*	2DT2138	2	>8/2		4/76

The antifungal activity of the lavender EOs studied was also tested against five fungal strains. The results in Table 10 showed that all fungal strains were inhibited (100%) with concentrations less than or equal to 3.13 μg/mL, except for the yeast *Candida dubliniensis* which showed remarkable resistance to *L. stoechas* EO where inhibition was achieved at a MIC of 6.25 μg/mL.

**TABLE 10:** MIC and MFC values (µg/mL) of the studied essential oils (µg/mL).

	*L. abrialis*		*L. stoechas*	
	MIC	MFC	MIC	MFC
*Candida albicans*	3.13	3.13	3.13	3.13
*Candida dubliniensis*	3.13	3.13	6.25	6.25
*Candida tropicalis*	3.13	6.25	3.13	6.25
*Candida parapsilosis*	3.13	6.25	3.13	6.25
*Aspergillus niger*	1.56	1.56	1.56	1.56

MFC, minimum fungicidal concentration.

### 3.6 Correlation between the chemical composition of the EOs studied and the antimicrobial activity

**FIGURE 9:**
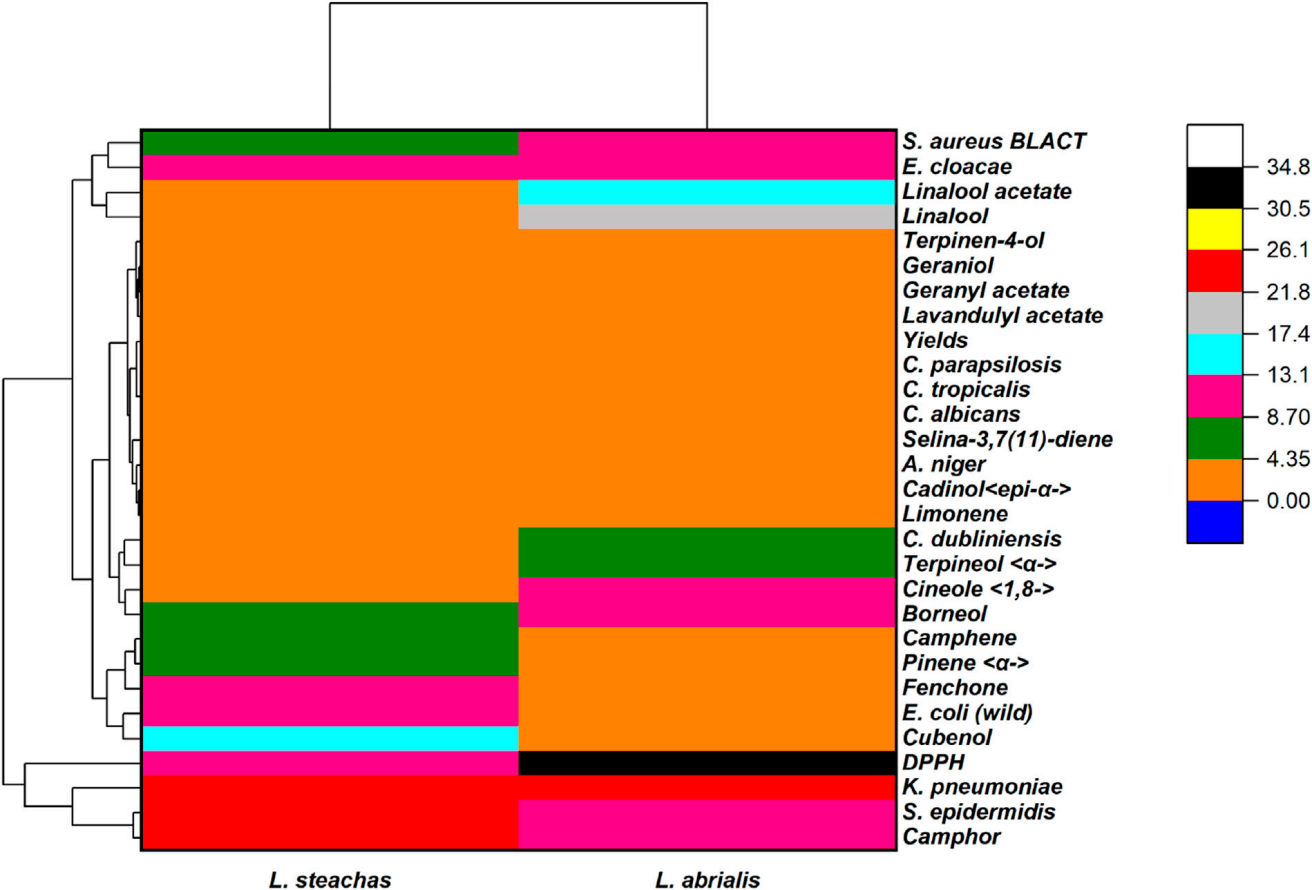
Heat map with a 2D cluster of the obtained results.

The Heat map is one of the most often used graphical representations, especially in the sciences, for illustrating the influence of phenomena, including organic ones (Ailli et al., 2023). This map was used in this case to ascertain the relationship between the chemical composition of the studied lavender EOs, the antioxidant activity, and the antimicrobial activity (Figure 9).

Oxygenated monoterpenes had a positive effect on bacterial and fungal strains. Camphor, as their major component, was more effective against *K. pneumoniae* and *S. epidermidis* while fenchone, pinene, and camphene have a stronger effect on *E. coli*. Regarding the inhibition of strain growth of *E. cloacae* and *S. aureus* BLACT, linalool displayed a very high positive correlation while the other compounds of the oxygenated monoterpene family have a moderate correlation with studied fungal strains. Borneol and cineol have a strong correlation with *C. dubliniensis*.

Oxygenated sesquiterpenes are more effective against bacterial strains except for cadinol which has a moderate effect against fungal strains. Indeed, cubenol which was the principal ingredient of *L. stoechas* essential oil had a strong correlation against *E. coli.* Likewise, linaool acetate, a primary constituent of *L. abrialis* EO, has a very substantial correlation with strain growth inhibition of *E. cloacae* and *S. aureus*. These results corroborate with previous studies including Radi et al. (2022) who showed that *L. stoechas* EOs demonstrated significant antibacterial activity against several strains, such as *S. aureus*, *Staphylococcus typhi*, *E. coli*, *A. baumanii*, *E. Cloacae* and *Staphylococcus dysenteria* and antifungal activity against *Candida glabrata*, *Candida albicans*, *Candida* spp., *Aspergillus fisheri,* and *Fusarum solani*. According to Dorman and Deans, (2000), the antibacterial activity was brought on by the presence of phenol (1,8 cineole), alcohols (cubenol, borneol, terpineol, *etc.*), aldehydes, and ketones (camphor, linalool acetate, *etc.*). Bachiri et al. (2016) reported that *L. stoechas* EOs showed an antibacterial action against the four pathogens *E. coli*, *K. pneumoniae*, *S. aureus*, and *P. mirabilis*. Moreover, N'Dédianhoua et al. (2014) discovered that *L. abrialis* EO has an antibacterial effect on *K. pneumoniae* and *E. coli*.

### 3.7 Molecular docking analysis

#### 3.7.1 *In silico* antibacterial activity

The *in vitro* analysis revealed that the extracts showed strong antibacterial activity against *S. aureus*, therefore we selected SarA protein of *S. aureus* for molecular docking analysis. The sarA locus regulates over 100 genes on the *S. aureus* chromosome, playing a role in the intrinsic multidrug resistance mechanism by regulating drug accumulation (O'Leary et al., 2004). This protein is considered a master regulator of biofilm formation in *S. aureus*, and targeting this protein has been explored by several researchers as a potential strategy for drug development (Arya et al., 2015; Hosen et al., 2023). Among the 56 phytochemicals from two extracts the top five best ligands were selected based on their lowest binding energy, interaction with target protein, pose and RMSD value shown in Table 11.

**TABLE 11:** The binding details of best five compounds against SarA protein (PDB:2fnp).

Complex	Binding energy (kcal/mol)	Amino acid residues	Bond types	Distance (Å)
2fnp + CID 15559789 (Ishwarone)	−9.8	A:Leu113	H	4.40
		A:Phe110	H	4.98
		B:Tyr142	H	5.19
		B:His159	H	4.89
2fnp + CID 10398656 (alpha-Cadinol)	−9.3	A:Leu113	H	3.76
		A:Phe110	H	5.32
		B:His159	H	5.50
		B:Leu160	Alkyl	3.56
2fnp + CID 86609 (alpha-Cubebene)	−8.6	A:Leu113	H	3.94
		B:Leu160	H	4.58
		B:His159	H	5.38
2fnp + CID 6429080 (Gleenol)	−6.5	A:Leu113	H	4.08
		B:Leu160	H	4.67
		A:Phe110	Alkyl	4.75
2fnp + CID 12302222 (epi-Cadinol)	−6.4	A:Val116	H	5.23
		B:Phe134	H	4.96
		B:Tyr162	Pi-Alkyl	5.37

**FIGURE 10:**
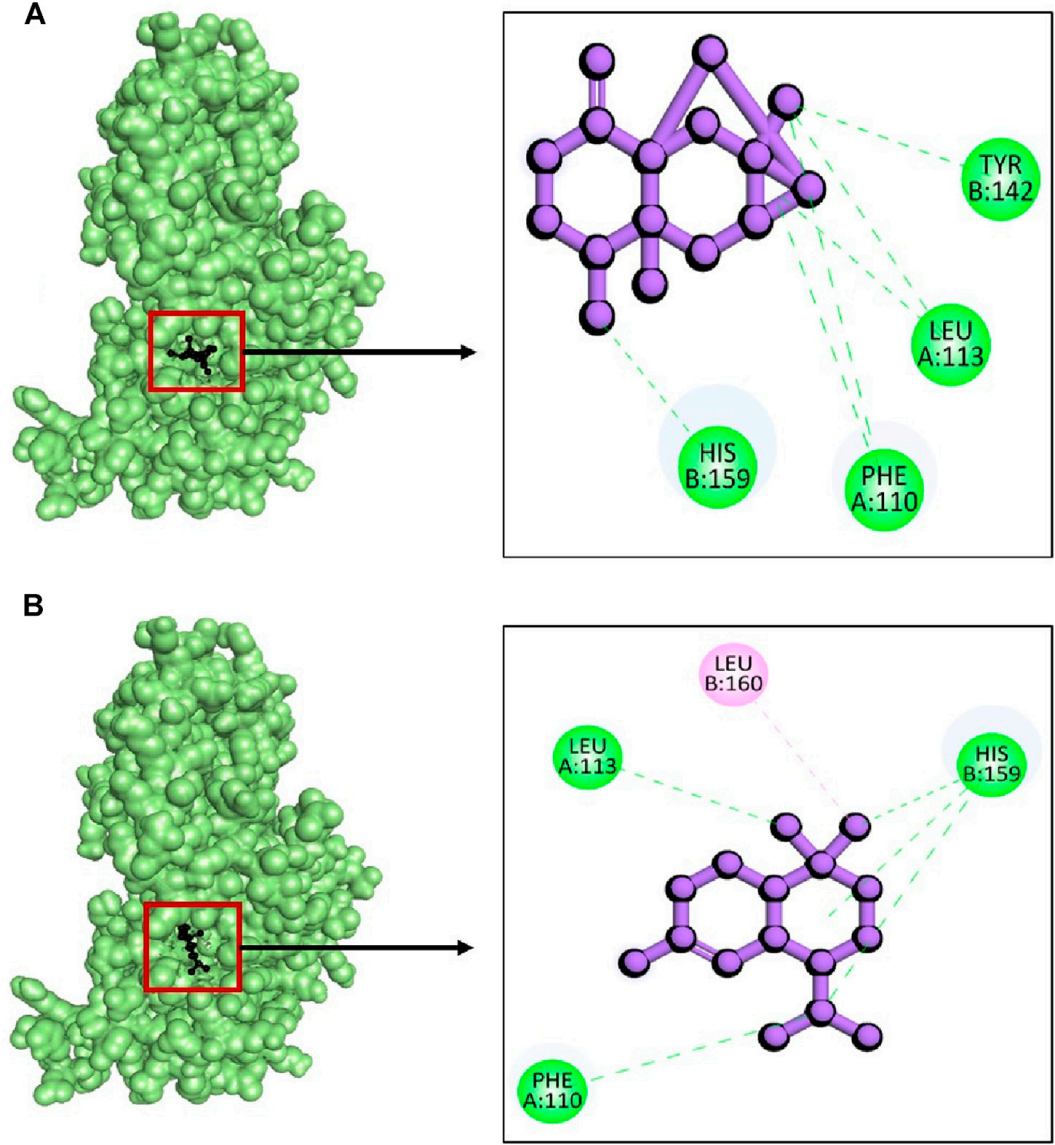
The interactions between phytochemicals and target SarA protein. Where **(A)** 2fnp + CID 15559789 and **(B)** 2fnp + CID 10398656.

Whereas, the compounds CID: 15559789 and CID: 10398656 exhibited maximum binding energy with −9.8 kcal/mol and 9.3 kcal/mol respectively (Table 1). The complex between 2fnp + CID: 15559789 was stabilized with highest four hydrogen bonds at A:Phe110, A:Leu113, B:Tyr142, and B:His159 (Figure 10A) while complex 2fnp + CID: 10398656 exhibited three hydrogen bond at A:Phe110, A:Leu113, and B:His159 (Figure 10B). The binding of these compounds at the active site of SarA protein could inhibit the pathogenesis of *S. aureus* (EF1).

#### 3.7.2 *In silico* antioxidant activity

This study also involved molecular docking study of NADPH oxidase (PDB: 2CDU) from *L. sanfranciscensis* to screen phytochemicals with the goal of identifying the most specific and potent inhibitors. NADPH oxidase is recognized for generating reactive oxygen species (ROS) at normal physiological levels. However, under certain disease conditions, it can become excessively activated, leading to the production of an excess of ROS. Several compounds have demonstrated the capacity to inhibit the overactivity of the enzyme (Kumar Deb et al., 2022; Kandsi et al., 2022). Interestingly, there limited published study has been reported on the inhibitory activity of *L. stoechas* and *L. abrialis* phytochemicals on NADPH oxidase. In the molecular docking study, from 56 selected phytochemical compounds of *L. stoechas* and *L. abrialis*, top 5 compounds were chosen based on the lowest binding energy (Table 12).

**TABLE 12:** The molecular interaction of plants phytochemicals against target NADPH oxidase (PDB: 2CDU).

Complex	Binding energy (kcal/mol)	Amino acid residues	Bond types	Distance (Å)
2CDU + CID 522296 (Selina-3,7 (11)-diene)	−10.8	A:Pro432	H	3.75
		B:His10	H	3.93
		B:Phe14	H	3.68
		B:Lys17	H	3.34
		B:Tyr62	H	4.23
		A:Phe433	Pi-Alkyl	5.25
		B:Val304	Pi-Alkyl	4.40
2CDU + CID 9855795 (Valencene)	−7.8	A:Lys17	H	4.21
		A:Phe14	H	4.44
		B:Phe433	H	4.94
		B:Ile438	H	4.97
		A:Tyr62	Alkyl	4.97
		A:Val304	Alkyl	5.39
		B:Pro432	Alkyl	4.50
2CDU + CID 94334 (Widdrol)	−7.9	A:PRO432	H	4.97
		A:PHE433	H	4.83
		B:HIS10	H	5.12
		B:PHE14	H	4.63
		B:LYS17	Pi-Alkyl	4.13
		B:TYR62	Pi-Alkyl	4.88
2CDU + CID 6429022 (trans-Calamenene)	−7.7	A:Pro432	H	4.17
		B:Tyr62	H	4.25
		B:Val304	H	4.79
		B:His10	Alkyl	4.70
		B:Phe14	Pi-Alkyl	5.23
		B:Tyr62	Pi-Alkyl	4.70
2CDU + CID 15559789 (Ishwarone)	−7.7	A:Phe443	H	4.69
		B:Lys17	H	3.90
		B:Phe14	H	4.96
		B:Tyr62	Alkyl	4.83

**FIGURE 11:**
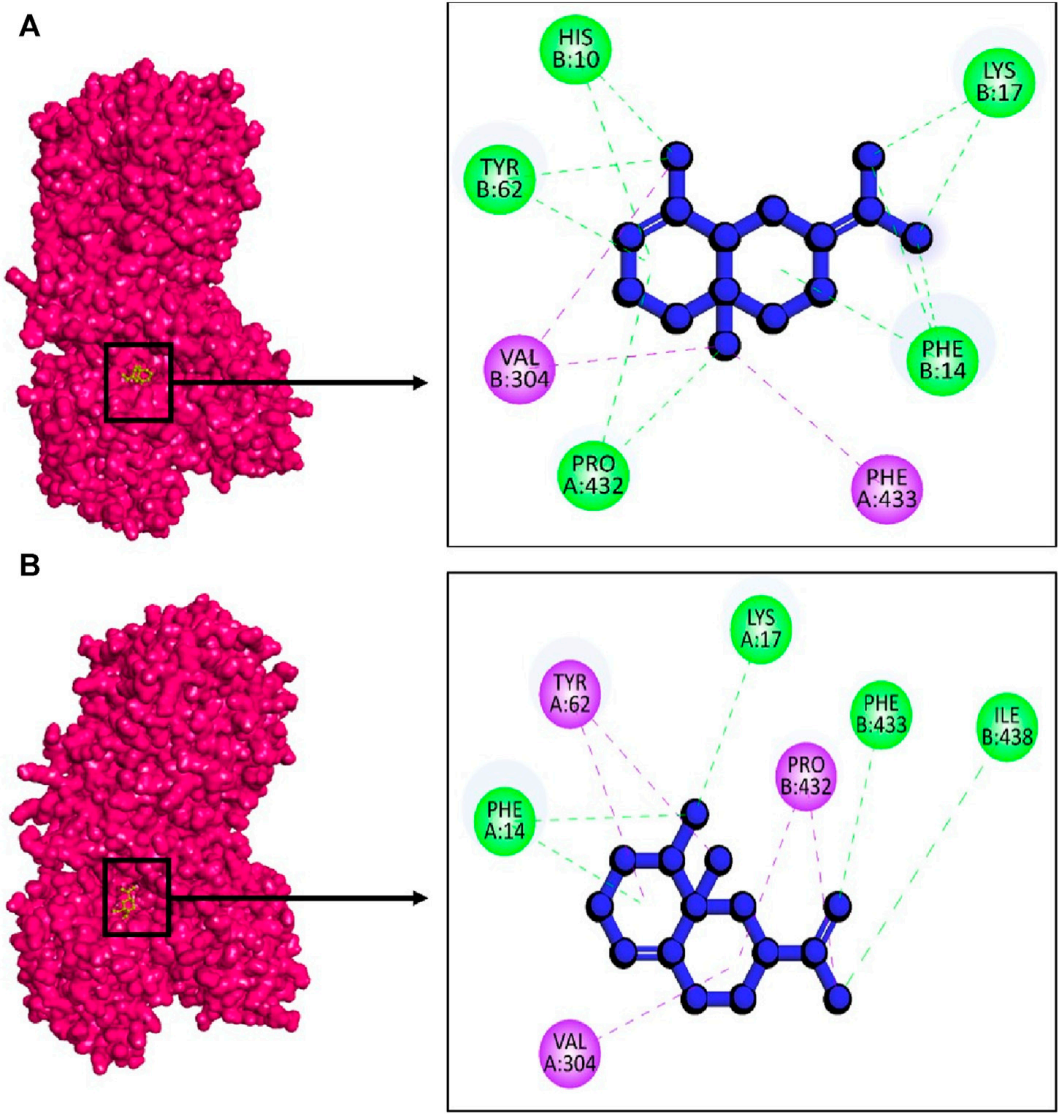
The interactions between phytochemicals and target NADPH oxidase. Where **(A)** 2CDU + CID 522296 and **(B)** 2CDU + CID 9855795.

In this case, against the target 2cdu, the highest binding energy was observed in the complex 2cdu + CID: 522296 with −10.8 kcal/mol followed by complex 2cdu + CID: 9855795 with binding energy −10.1 kcal/mol. The docking results revealed that to inhibit the 2cdu, the compound CID: 522296 showed five hydrogen bond at A:Pro432, B:His10, B:Phe14, B: Lys17, and B:Tyr62 (Figure 11A). Similarly, the complex 2cdu + CID: 9855795 stabilized with target protein with three hydrogen bond at A:Phe14, A:Lys17, B:Phe433, and B:Ile438 (Figure 11B).

## 4 Conclusion

The purpose of this study was to compare the chemical profile, antioxidant activity, and antimicrobial activity of essential oils extracted from two species: cultivated lavender (*L. abrialis*) and wild lavender (*L.stoechas*) as part of the valorization of aromatic and medicinal plants in the commune of Oulmès in Morocco.

Results of hydrodistillation extractions from the aerial parts of the two lavender species under study revealed that *L. abrialis* produced more essential oil than *L. stoechas*. According to the analysis of the EOs’ chemical composition, these two lavenders were particularly high in terpenes and contained a variety of typical chemical components, albeit in varying amounts. Linalool, linalyl acetate, and camphor represented most of the chemical constituents of *L. abrialis*, whereas cubenol, fenchone, and camphor make up the principal components of *L. stoechas*.

These terpene molecules in EOs contribute to their intriguing antioxidant and antibacterial effects. In this present work, *L. stoechas* was found to have greater antioxidant activity than *L. abrialis*. Furthermore, the investigated EOs had strong antibacterial activity against yeast, mold, and bacteria strains (both Gram-positive and Gram-negative). Because of these proprieties as natural antibacterial and antifungal agents, these oils are extremely valuable as a source of phytopharmaceutical ingredients that can be used to treat both bacterial and fungal diseases. The acquired results allowed us to conduct future experiments to develop formulations for using these bioactive natural compounds for their antioxidant, antibacterial, and antifungal characteristics in the food, pharmaceutical, and cosmetic industries.

## Data Availability

The raw data supporting the conclusion of this article will be made available by the authors, without undue reservation.
